# A Self-Assembling Peptide Platform for Intratumoral Doxorubicin Delivery and Preliminary Immune-Related Modulation in B16-F10 Melanoma

**DOI:** 10.3390/biomedicines14071624

**Published:** 2026-07-19

**Authors:** Xufang Ying, Jingjing Peng, Zhiqing Ben, Xiaoyan Bao, Linjie Wu, Xin Tan, Xiaoyan Sun, Yufan Yang, Yiqing Shen, Zhicheng Zhang, Ruolin Jiang, Yaxin Qin, Lin Zhou, Min Han, Shugang Yang

**Affiliations:** 1 Department of Gastrointestinal Surgery 2 Section, Institute of Abdominal Surgery, The First Affiliated Hospital, Fujian Medical University, 20th, Chazhong Road, Fuzhou 350005, China; 2 Institute of Pharmaceutics, School of Pharmacy, Zhejiang University, Hangzhou 310058, China; 22360551@zju.edu.cn (X.Y.); 3220104170@zju.edu.cn (J.P.); 22319129@zju.edu.cn (Z.B.); baoxiaoyan@zju.edu.cn (X.B.); 22119114@zju.edu.cn (L.W.); tanxin@zju.edu.cn (X.T.); xiaoyansun@zju.edu.cn (X.S.); 22419047@zju.edu.cn (Y.Y.); 22419093@zju.edu.cn (Y.S.); 22260510@zju.edu.cn (R.J.); 3170105890@zju.edu.cn (Y.Q.); 22519065@zju.edu.cn (L.Z.); 3 Polytechnic Institute, Zhejiang University, Hangzhou 310058, China; 4 Taizhou Institute of Zhejiang University, Taizhou 318000, China; 5 The Second Affiliated Hospital, Zhejiang University School of Medicine, Zhejiang University, Hangzhou 310058, China; zhicheng_zhang@zju.edu.cn; 6 Jinhua Institute of Zhejiang University, Jinhua 321299, China; 7 National Regional Medical Center, Binhai Campus of the First Affiliated Hospital, Fujian Medical University, Fuzhou 350004, China

**Keywords:** self-assembling peptide, CD3-recognition peptide, local drug delivery, doxorubicin, melanoma, chemo-immunomodulation

## Abstract

**Background:** Local drug delivery can increase antitumor exposure while limiting systemic toxicity, but chemotherapy-only local treatment may not fully control residual tumor growth in immunosuppressive tumor microenvironments. This study aimed to develop and preliminarily evaluate ffky-antiCD3, a CD3-recognition peptide-functionalized self-assembling peptide platform for intratumoral doxorubicin (DOX) delivery. **Methods:** The Nap aromatic group in a previous Nap-ffky scaffold was removed to improve aqueous dispersibility, and the CD3-recognition sequence AKMGEGGWGANDY was introduced to generate ffky-antiCD3. The peptide/formulation was characterized by reversed-phase high-performance liquid chromatography, mass spectrometry, TEM, circular dichroism spectroscopy, and a preliminary in vitro DOX release assay under tumor-mimicking acidic conditions. Antitumor efficacy, tumor histopathology, image-based CD3/CD8 semi-quantification, splenic IFN-γ levels, serum biochemistry, organ coefficients, and major-organ histology were assessed after repeated intratumoral treatment in B16-F10 melanoma-bearing C57BL/6 mice. **Results:** ffky-antiCD3 formed assemblies with a β-sheet-rich secondary structure. TEM observation further showed heterogeneous irregular/network-like supramolecular assemblies, and the preliminary release assay suggested slower apparent DOX release from ffky-antiCD3/DOX than from free DOX at pH 6.5. Among the tested groups, ffky-antiCD3/DOX produced the strongest short-term tumor-growth inhibition and the lowest endpoint tumor weight during the 10-day observation period. Ki67 staining decreased, and TUNEL signals increased after ffky-antiCD3/DOX treatment, supporting reduced proliferation and enhanced apoptosis-related damage. CD3/CD8 staining and exploratory splenic IFN-γ measurements indicated preliminary immune-related changes associated with ffky-antiCD3-containing formulations. Body weight, organ weights, serum biochemical markers, and major-organ H&E staining revealed no obvious short-term toxicity signals under the tested regimen. **Conclusions:** ffky-antiCD3/DOX represents a candidate local peptide-based chemo-immunomodulatory formulation. Its immune mechanism, release behavior, biodistribution, and long-term efficacy and safety require further validation before strong mechanistic or translational claims are made.

## 1. Introduction

Surgery, chemotherapy, radiotherapy, and immunotherapy remain major components of cancer treatment, yet local recurrence and residual tumor growth continue to limit clinical outcomes [1,2]. For solid tumors such as melanoma, incomplete eradication of residual lesions remains an important clinical challenge [3], and pro-tumorigenic remodeling of the local microenvironment can further promote disease progression [4,5,6,7]. Local delivery systems have therefore attracted increasing attention because they can concentrate therapeutics at the tumor site, reduce off-target exposure, and provide a platform for combining cytotoxic and microenvironment-modulating functions [8,9,10,11].

Doxorubicin (DOX) is a widely used chemotherapeutic drug with potent antitumor activity [12]. Nevertheless, systemic administration is associated with dose-limiting toxicities, and free drug can be rapidly cleared from local tissues [13,14]. Peptide-based supramolecular assemblies provide a modular strategy for local drug delivery because their sequences can be engineered to modulate assembly behavior, drug association, degradation, and biological interactions [15,16,17,18,19,20,21]. In previous local-delivery work, Nap-ffky/DOX used a Nap aromatic hydrophobic group to support stable assembly and local drug retention. While that design is suitable for depot formation, excessive hydrophobicity may restrict dispersion and tissue distribution during repeated intratumoral administration.

In addition to direct tumor-cell killing, immune engagement is increasingly recognized as a key factor in local tumor control [4,5,6,7]. CD3 is expressed on T cells and has been widely used as a target in T-cell redirecting or activating strategies [22,23,24]. A peptide sequence previously reported to recognize CD3, AKMGEGGWGANDY, may therefore provide a potential functional interface for interacting with T cells [25]. However, whether the incorporation of such a sequence into a local self-assembling peptide delivery system can improve antitumor efficacy and alter immune cell infiltration remains to be experimentally validated.

In this study, we constructed ffky-antiCD3 by removing the Nap group from Nap-ffky and introducing the previously reported CD3-recognition peptide sequence at the terminus. The resulting peptide was combined with DOX to generate a DOX-associated ffky-antiCD3/DOX local formulation. We evaluated its chemical structure, secondary structure, assembly morphology, preliminary DOX release behavior, short-term antitumor efficacy, tumor histopathology, preliminary immune-related changes, and short-term tolerability in a B16-F10 melanoma model. The present manuscript frames the current findings as exploratory proof-of-concept evidence while reserving mechanistic immune, biodistribution, and long-term efficacy claims for subsequent validation.

## 2. Materials and Methods

### 2.1. Materials, Cells, and Animals

Phosphate-buffered saline (PBS) powder was purchased from Wuhan Boster Biological Technology Co., Ltd. (Wuhan, China). Dulbecco’s modified Eagle’s medium (DMEM; high glucose), penicillin–streptomycin solution, and trypsin containing 0.02% EDTA were obtained from Shanghai Yeasen Biotechnology Co., Ltd. (Shanghai, China). Cell-grade dimethyl sulfoxide (DMSO) was purchased from Beyotime Biotechnology Co., Ltd. (Shanghai, China), and fetal bovine serum was obtained from Gibco (Waltham, MA, USA). Tumor tissue hematoxylin and eosin (H&E) staining, Ki67 immunostaining, TUNEL staining, serum biochemical and routine blood analyses, and major-organ histological staining were performed by Hangzhou Fuyou Biotechnology Co., Ltd. (Hangzhou, China).

B16-F10 mouse melanoma cells were obtained from the Cell Resource Center of the Shanghai Institutes for Biological Sciences, Chinese Academy of Sciences (Shanghai, China). Male SPF C57BL/6 mice aged 5 weeks were purchased from Hangzhou Qizhen Laboratory Animal Technology Co., Ltd. (Hangzhou, China).

The key data-generating instruments used in this study included an LCMS-2020 liquid chromatography–mass spectrometry system (Shimadzu Corporation, Kyoto, Japan) for peptide structural confirmation, and a J-1700 circular dichroism spectrometer (JASCO Corporation, Tokyo, Japan) for secondary-structure characterization. Cell culture was performed using a CO_2_ incubator (HF90/HF240; Shanghai Lishen Scientific Instrument Co., Ltd., Shanghai, China), and ultrapure water was prepared using a Milli-Q ultrapure water system (Millipore, Billerica, MA, USA).

### 2.2. Synthesis and Structural Confirmation of ffky-antiCD3

The ffky-antiCD3 peptide was synthesized by standard Fmoc solid-phase peptide synthesis and purified by reversed-phase high-performance liquid chromatography (RP-HPLC). The peptide structure was verified by mass spectrometry using an LCMS-2020 system (Shimadzu Corporation, Kyoto, Japan) equipped with electrospray ionization. For HPLC analysis, a Sinochrom ODS-BP 5 column (250 × 4.6 mm) (Dalian Elite Analytical Instruments Co., Ltd., Dalian, China) was used. Mobile phase A consisted of 0.1% trifluoroacetic acid in acetonitrile, and mobile phase B consisted of 0.1% trifluoroacetic acid in water. The gradient was 22% A/78% B at 0.01 min, 47% A/53% B at 20.0 min, and 100% A/0% B at 20.1 min, followed by termination at 25.0 min. The flow rate was 1.0 mL/min, the detection wavelength was 220 nm, and the injection volume was 5 µL.

### 2.3. Preparation of ffky-antiCD3/DOX Formulation

The ffky-antiCD3/DOX formulation was prepared by mixing DOX with ffky-antiCD3 at a molar ratio of n(DOX):n(ffky-antiCD3) = 0.05.

### 2.4. Circular Dichroism Spectroscopy

Circular dichroism (CD) spectra of ffky-antiCD3 aqueous solution (0.2 mg/mL) were collected using a J-1700 CD spectrometer over the wavelength range of 190–260 nm at a scanning speed of 100 nm/min. The solvent background was subtracted, and secondary-structure fractions were estimated by spectral fitting.

### 2.5. Intratumoral Treatment in B16-F10 Tumor-Bearing Mice

All animal procedures were performed in accordance with the requirements of the AILIMO Laboratory Animal Welfare and Ethics Committee (approval number: ALM20250816). B16-F10 tumor-bearing C57BL/6 mice were randomly assigned to four groups when tumor volume reached approximately 50 mm^3^: Control, DOX, ffky-antiCD3, and ffky-antiCD3/DOX. The planned sample size was *n* = 6 per group for tumor-growth analysis. The control group received PBS by intratumoral injection. The DOX group received free DOX at 4 mg/kg. The ffky-antiCD3 group received the peptide formulation at a volume matched to the drug-loaded formulation. The ffky-antiCD3/DOX group received the peptide/DOX formulation at a DOX dose of 4 mg/kg, with n(DOX):n(ffky-antiCD3) = 0.05. Injections were administered on Days 0, 3, and 7. Tumor length (L) and width (W) were measured every two days with calipers, and tumor volume was calculated as V = (L × W^2^)/2. Body weight and general condition were recorded throughout the experiment. Mice were euthanized on Day 10, and tumors and major organs were collected for analysis.

### 2.6. Histological and Immunological Analyses

Tumor tissues were processed for H&E staining, Ki67 immunostaining, and TUNEL fluorescence staining to evaluate tissue morphology, proliferative activity, and apoptosis-related DNA fragmentation, respectively. CD3/CD8 double immunofluorescence staining was used to assess preliminary T-cell-related changes in tumor tissues. For image-based semi-quantification, CD3+ and CD8+ cells were counted in multiple non-overlapping tumor regions from the available stained sections using ImageJ 1.54g, and the values were normalized to DAPI+ nuclei and expressed as positive cells/DAPI+ cells (%). Spleen samples were collected to measure IFN-γ secretion as an exploratory splenic cytokine endpoint. Major organs including the heart, liver, spleen, lung, and kidney were collected, weighed, and examined by H&E staining. Serum biochemistry markers including ALT, AST, TP, ALB, CREA, and CK-MB were analyzed to evaluate short-term tolerability.

### 2.7. Statistical Analysis

Some of the graphs were plotted with the aid of Origin 2021. GraphPad Prism 9 was used for statistical analysis. Data are expressed as mean ± standard deviation (SD), with exact n values indicated in the corresponding figure legends. One-way analysis of variance (ANOVA) followed by Tukey’s post hoc test was applied for comparisons involving one independent variable. A two-way ANOVA followed by Bonferroni’s post hoc test was applied, where two independent variables were involved. Independent-sample *t* tests were used for two-group comparisons. Before applying ANOVA-based comparisons, data distribution and variance assumptions were checked using GraphPad Prism and graphical inspection; because some exploratory assays had small sample sizes, these results were interpreted cautiously. Randomization was applied for animal grouping, and image-based quantification was performed using the same thresholding and counting rules across all groups. A *p* value of less than 0.05 was considered statistically significant.

## 3. Results

### 3.1. Design Rationale and Structural Confirmation of ffky-antiCD3

**Figure 1:**
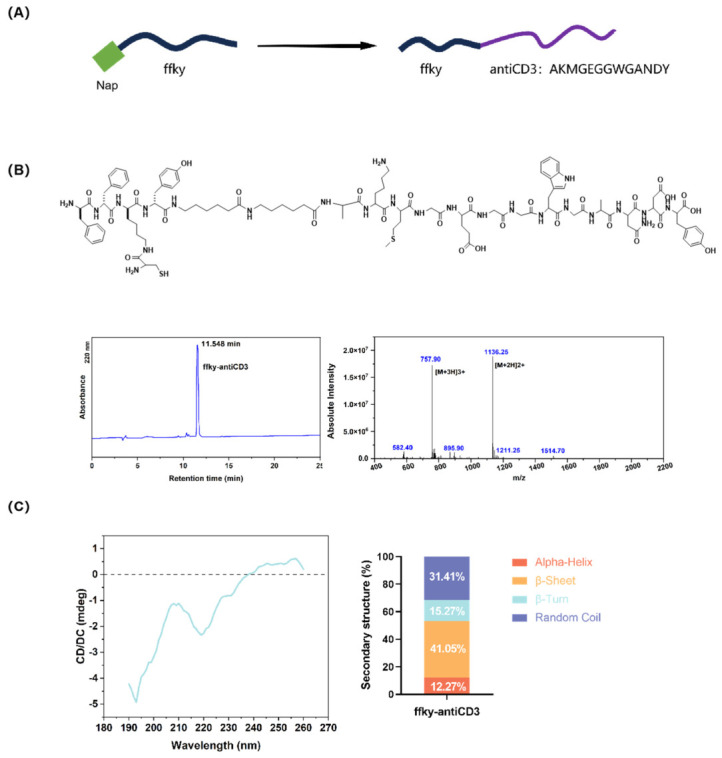
Design and physicochemical characterization of ffky-antiCD3. (**A**) Structural modification strategy from Nap-ffky to ffky-antiCD3 by removing the Nap aromatic hydrophobic group and introducing the CD3-recognition peptide sequence AKMGEGGWGANDY. (**B**) Chemical structure, RP-HPLC chromatogram, and MS spectrum of ffky-antiCD3. (**C**) CD spectrum and fitted secondary-structure fractions, indicating a β-sheet-rich conformation.

The previous Nap-ffky/DOX system relied on the Nap aromatic hydrophobic group to drive self-assembly and maintain a local drug reservoir. In the present design, the Nap group was removed to reduce hydrophobicity and improve dispersion during repeated local administration. The CD3-recognition sequence AKMGEGGWGANDY was introduced at the peptide terminus to create ffky-antiCD3, which was intended to retain peptide self-assembly while providing a potential T-cell-interactive interface (Figure 1A). This design rationale should be interpreted as a functional hypothesis rather than direct proof of CD3-dependent activity.

RP-HPLC analysis showed a dominant peak at approximately 11.548 min, suggesting good purity of the synthesized peptide. Mass spectrometry showed multicharged ion peaks corresponding to a calculated molecular weight of approximately 2270.5–2270.7 Da, which matched the expected molecular weight of the target peptide (Figure 1B). These data confirmed successful synthesis and structural reliability of ffky-antiCD3.

### 3.2. ffky-antiCD3 Forms β-Sheet-Rich Assemblies and Modulates DOX Release Behavior

CD spectroscopy revealed a distinct conformational profile between 190 and 260 nm. Secondary-structure fitting suggested that ffky-antiCD3 consisted mainly of β-sheet (approximately 41.05%) and random coil (approximately 31.41%) structures, with smaller fractions of β-turn (approximately 15.27%) and α-helix (approximately 12.27%) components (Figure 1C).

To further evaluate the assembly morphology, we performed TEM observation according to Method S1. Representative TEM images showed that ffky-antiCD3/DOX formed heterogeneous supramolecular assemblies with irregular/network-like features rather than uniform spherical nanoparticles (Figure S1). This morphology was consistent with the β-sheet-rich conformational characteristics observed by CD spectroscopy and supported the assembly behavior of the peptide-based formulation.

To preliminarily assess whether peptide association affected DOX diffusion, an in vitro release assay was performed according to Method S2 under a tumor-mimicking acidic condition containing 10% FBS. Compared with free DOX, ffky-antiCD3/DOX showed a slower apparent DOX release profile at pH 6.5, characterized by an initial release followed by a relatively plateaued phase during the observation period (Figure S2). These results suggest that association with the peptide assemblies may partially retard DOX diffusion under these in vitro conditions.

### 3.3. ffky-antiCD3/DOX Shows Improved Antitumor Efficacy After Repeated Intratumoral Administration

**Figure 2:**
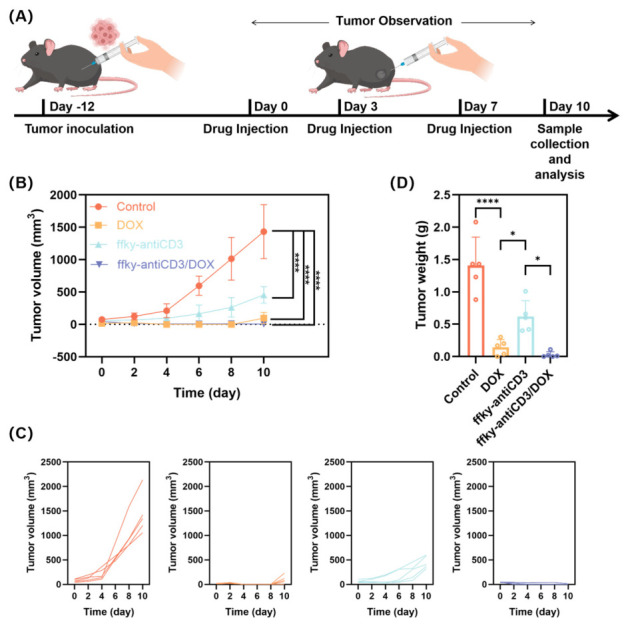
In vivo antitumor efficacy of the ffky-antiCD3/DOX local delivery system. (**A**) Treatment schedule for intratumoral injections in B16-F10 tumor-bearing C57BL/6 mice. (**B**) Average tumor growth curves. (**C**) Individual tumor growth curves. (**D**) Endpoint tumor weights at Day 10. Data are presented as mean ± SD; *n* = 6; * *p* < 0.05, **** *p* < 0.0001.

The antitumor effect of ffky-antiCD3/DOX was evaluated in a B16-F10 melanoma-bearing mouse model using three intratumoral injections on Days 0, 3, and 7 (Figure 2A). The control group showed rapid tumor progression. Free DOX significantly inhibited tumor growth, while ffky-antiCD3 alone also showed a moderate inhibitory tendency compared with the control group. The ffky-antiCD3/DOX group maintained the smallest tumor volume throughout the observation period (Figure 2B,C).

At the Day 10 endpoint, tumor weight was lowest in the ffky-antiCD3/DOX group (Figure 2D). These findings indicate that the DOX-associated peptide formulation produced the strongest short-term antitumor effect among the tested treatments during the 10-day observation period. This enhanced effect may reflect the combined influence of local DOX delivery and the peptide-based formulation context.

### 3.4. Tumor Histopathology Supports Reduced Proliferation and Increased Apoptosis-Related Damage

**Figure 3:**
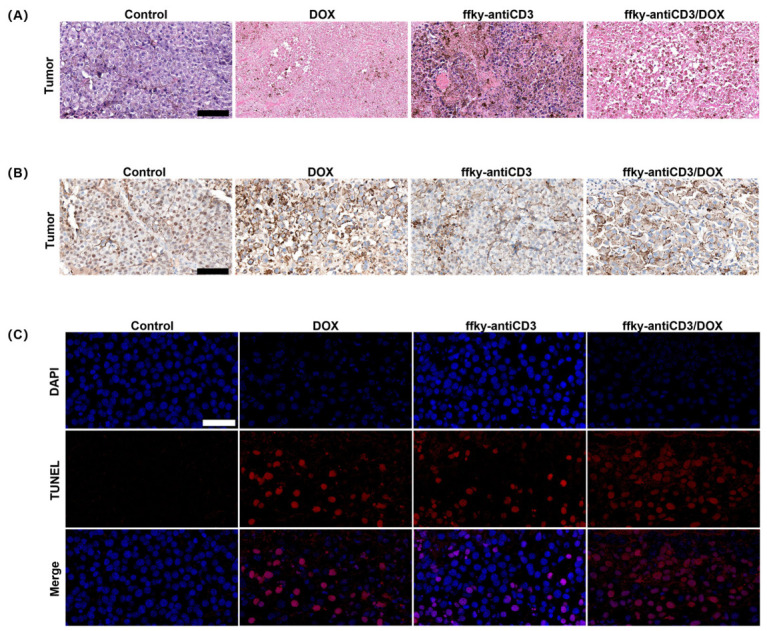
Histopathological assessment of tumor tissues. (**A**) Representative H&E-stained tumor sections. Scale bar: 100 µm. (**B**) Ki67 immunostaining showing tumor cell proliferative activity. Scale bar: 100 µm. (**C**) TUNEL staining showing apoptosis-related DNA fragmentation. TUNEL, red; DAPI, blue; scale bar: 50 µm.

H&E staining showed densely arranged tumor cells with hyperchromatic nuclei in the control group, consistent with highly proliferative tumor morphology. DOX and ffky-antiCD3/DOX treatments induced more evident tissue disruption and degenerative or necrotic regions, while the ffky-antiCD3 group showed intermediate pathological changes (Figure 3A).

Ki67 immunostaining revealed extensive positivity in control tumors, indicating active proliferation. The DOX group displayed decreased Ki67 staining, and the ffky-antiCD3 group also showed a reduction compared with the control group. The ffky-antiCD3/DOX group exhibited the most pronounced Ki67 reduction (Figure 3B). TUNEL staining showed weak apoptotic signals in the control group, increased signals in the DOX and ffky-antiCD3 groups, and the strongest TUNEL-positive tendency in the ffky-antiCD3/DOX group (Figure 3C). Together, these histological findings support the tumor-growth inhibition observed in vivo.

### 3.5. CD3/CD8 Staining and Splenic IFN-γ Provide Preliminary Immune-Related Observations

**Figure 4:**
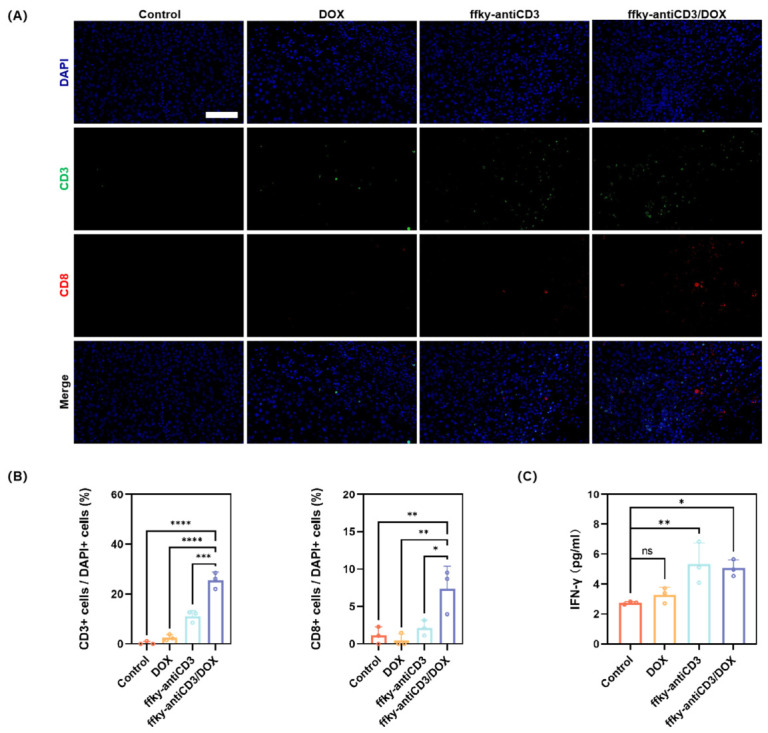
Preliminary immune-related observations after local treatment. (**A**) Representative CD3/CD8 immunofluorescence staining of tumor sections. DAPI, blue; CD3, green; CD8, red; scale bar: 100 µm. (**B**) Image-based semi-quantification of CD3+ cells/DAPI+ cells and CD8+ cells/DAPI+ cells in tumor sections. (**C**) IFN-γ levels in spleen samples. Data are presented as mean ± SD; exact n values are indicated for each assay; ns, not significant; * *p* < 0.05; ** *p* < 0.01; *** *p* < 0.001; **** *p* < 0.0001.

CD3/CD8 double immunofluorescence staining was used to explore whether the CD3-recognition peptide-containing formulation was associated with local T-cell-related changes. Control tumors showed weak CD3+ and CD8+ signals, and free DOX alone did not clearly enhance these signals. In contrast, ffky-antiCD3 treatment increased CD3+ staining with detectable CD8+ staining, while ffky-antiCD3/DOX produced the most pronounced CD3+/CD8+ fluorescence signals in some tumor regions (Figure 4A). Image-based semi-quantification further showed higher percentages of CD3+ cells/DAPI+ cells and CD8+ cells/DAPI+ cells in the ffky-antiCD3/DOX group than in the control, DOX, and ffky-antiCD3 groups (Figure 4B). Because the available histological material was limited, this quantification was used as supportive exploratory evidence rather than definitive proof of immune-cell recruitment or activation.

Splenic IFN-γ levels were also measured as an exploratory cytokine endpoint. IFN-γ levels were higher in the ffky-antiCD3 and ffky-antiCD3/DOX groups than in the control group, whereas free DOX did not differ significantly from the control group (Figure 4C). These findings suggest that ffky-antiCD3-containing formulations may be associated with preliminary immune-related responses. However, because this study did not include flow cytometry, T-cell activation assays, cytokine panels, blocking studies, scrambled peptide controls, or depletion experiments, these data should be interpreted as preliminary immune-related observations rather than definitive mechanistic proof of CD3-mediated immune activation.

### 3.6. Repeated Intratumoral Injection Shows No Obvious Short-Term Toxicity Signals Under the Tested Regimen

**Figure 5:**
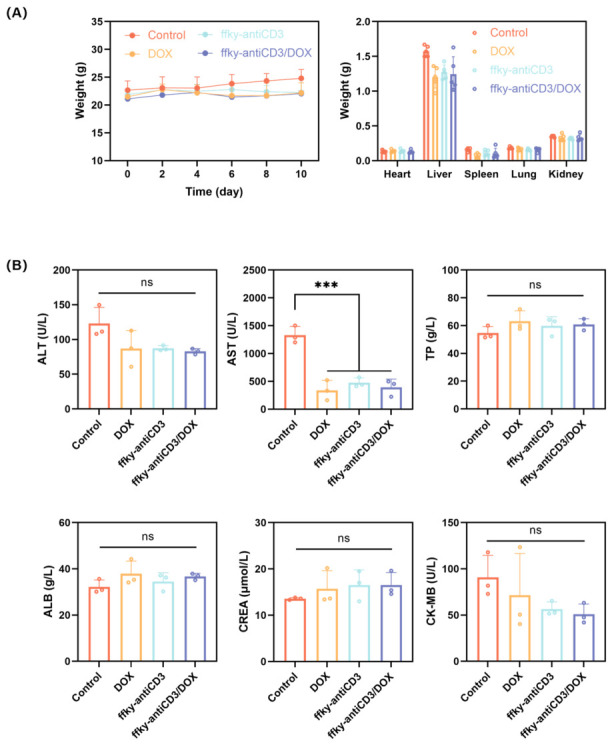
Short-term tolerability and serum biochemistry after intratumoral injections. (**A**) Body-weight changes over the observation period and endpoint major-organ weights. (**B**) Serum biochemistry indexes, including ALT, AST, TP, ALB, CREA, and CK-MB. Data are presented as mean ± SD; *n* = 3 for serum biochemistry and endpoint organ-weight analyses; ns, not significant; *** *p* < 0.001.

Body weights remained generally stable from Day 0 to Day 10 in all groups, suggesting that the three-dose intratumoral treatment regimen was tolerated during the short observation period (Figure 5A). Endpoint weights of the heart, liver, spleen, lung, and kidney did not show obvious abnormal enlargement or atrophy among groups. Serum biochemistry showed no significant differences in ALT, TP, ALB, CREA, or CK-MB. AST was higher in the control group than in the treatment groups, which may be related to greater tumor burden and systemic stress rather than treatment-induced liver injury (Figure 5B).

**Figure 6:**
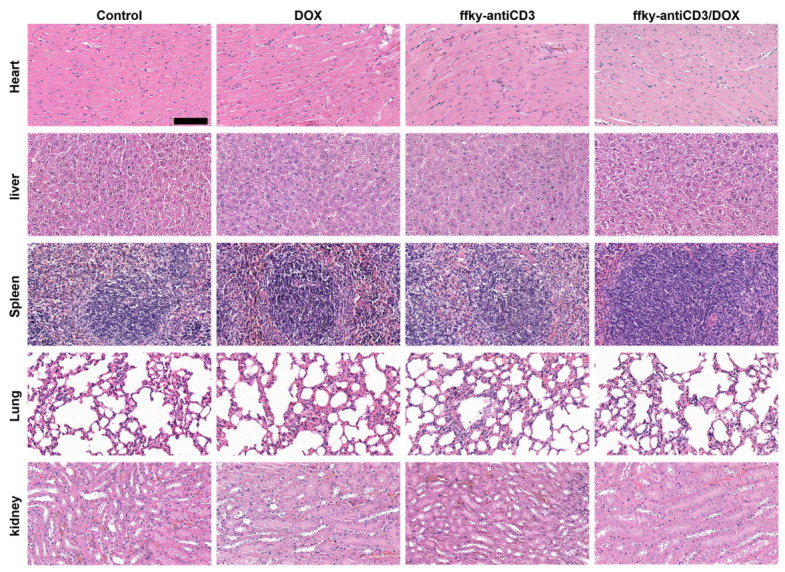
Representative H&E-stained images of major organs. H&E staining of heart, liver, spleen, lung, and kidney sections from different treatment groups. Scale bar: 100 µm.

H&E staining of major organs showed generally preserved tissue architecture without obvious inflammatory infiltration, necrosis, or structural disruption (Figure 6). These results indicate that ffky-antiCD3 and ffky-antiCD3/DOX showed no obvious short-term toxicity signals under the tested dose, intratumoral dosing schedule, and 10-day observation period. The current safety data should not be generalized to long-term or repeated administration safety without further toxicological evaluation.

## 4. Discussion

This study shows that a peptide sequence previously reported to recognize CD3 can be incorporated into a self-assembling peptide local formulation for DOX-associated intratumoral treatment. Compared with free DOX and peptide alone, ffky-antiCD3/DOX produced the strongest short-term tumor-growth inhibition in the B16-F10 model during the 10-day observation period. Histological results supported this therapeutic effect by showing reduced proliferation and increased apoptosis-related damage in tumor tissues.

The design differs from a depot-oriented Nap-ffky system by reducing hydrophobicity and introducing a functional peptide sequence intended to provide a potential T-cell-interactive interface. CD results indicate that ffky-antiCD3 still forms β-sheet-rich assemblies after removal of the Nap group, suggesting that the ffky backbone and CD3-recognition extension can provide molecular interactions for supramolecular organization. However, the present data do not directly establish intratumoral retention, altered biodistribution, reduced systemic exposure, or in vivo release behavior. These issues require dedicated quantitative studies with multiple time points and validated analytical methods.

The increased CD3+/CD8+ staining, image-based semi-quantification, and exploratory splenic IFN-γ results suggest that ffky-antiCD3-containing formulations were associated with preliminary immune-related changes. Nevertheless, the current evidence is not sufficient to claim a fully established CD3-mediated immunotherapeutic mechanism. Future studies should include flow cytometric profiling of tumor-infiltrating lymphocytes, T-cell activation and exhaustion markers, cytokine panels, physicochemically matched scrambled peptide controls, direct T-cell binding assays, CD3-blocking or depletion experiments, and longer observation windows. These experiments would clarify whether the short-term antitumor benefit is driven mainly by local DOX exposure, release behavior, peptide-intrinsic effects, immune engagement, or a combination of these factors.

From a translational perspective, intratumoral delivery is increasingly used for local immune and drug-delivery interventions, and recent delivery systems such as biomimetic nanovesicles and engineered lipid vesicles further illustrate the importance of matching delivery design with tumor biology [26,27]. A modular peptide platform that combines chemotherapy with a potential immune-interactive interface could be useful for accessible tumors or postoperative residual lesions. However, this preliminary study remains limited by the short 10-day observation period, small sample sizes for some safety and cytokine assays, absence of formal survival or recurrence monitoring, lack of quantitative release and biodistribution data, and absence of detailed mechanistic immunology. These limitations should be addressed in future studies before claims of durable tumor control, systemic immune activation, or long-term safety are made.

## 5. Conclusions

We constructed ffky-antiCD3 by introducing a peptide sequence previously reported to recognize CD3 into a self-assembling ffky backbone and used it to prepare a DOX-associated local formulation. The peptide was successfully synthesized and showed β-sheet-rich conformational features. In the B16-F10 melanoma model, repeated intratumoral administration of ffky-antiCD3/DOX produced the strongest short-term tumor-growth inhibition, reduced Ki67 staining, increased TUNEL signals, and was associated with exploratory increases in CD3+/CD8+ staining and splenic IFN-γ levels. No obvious short-term toxicity signals were detected under the tested conditions. Overall, ffky-antiCD3/DOX is a candidate local chemo-immunomodulatory peptide formulation, although release behavior, biodistribution, CD3 dependence, durability of efficacy, and long-term safety require further validation before strong immunotherapeutic or translational claims can be made.

## Data Availability

The data that support the findings of this study are available from the corresponding authors upon reasonable request.

## Supplementary Materials

The following supporting information can be downloaded at: https://www.mdpi.com/article/10.3390/biomedicines14071624/s1, Method S1. Transmission electron microscopy (TEM); Method S2. In vitro release of doxorubicin; Figure S1. Representative TEM images of ffky-antiCD3/DOX assemblies; Figure S2. Preliminary in vitro DOX release behavior under tumor-mimicking acidic conditions.

## Abbreviations

The following abbreviations are used in this manuscript:

ALB	Albumin
ALT	Alanine aminotransferase
ANOVA	Analysis of variance
APC	Article processing charge
AST	Aspartate aminotransferase
CD	Circular dichroism
CD3	Cluster of differentiation 3
CD8	Cluster of differentiation 8
CK-MB	Creatine kinase-MB
CO_2_	Carbon dioxide
CREA	Creatinine
DAPI	4′,6-diamidino-2-phenylindole
DMEM	Dulbecco’s modified Eagle’s medium
DMSO	Dimethyl sulfoxide
DNA	Deoxyribonucleic acid
DOX	Doxorubicin
EDTA	Ethylenediaminetetraacetic acid
Fmoc	9-fluorenylmethoxycarbonyl
H&E	Hematoxylin and eosin
HPLC	High-performance liquid chromatography
IFN-γ	Interferon-gamma
LCMS	Liquid chromatography–mass spectrometry
MS	Mass spectrometry
Nap	Naphthalene
PBS	Phosphate-buffered saline
RP-HPLC	Reversed-phase high-performance liquid chromatography
SD	Standard deviation
SPF	Specific-pathogen-free
TP	Total protein
TUNEL	Terminal deoxynucleotidyl transferase dUTP nick-end labeling
