# Supplementary material for: A Self-Assembling Peptide Platform for Intratumoral Doxorubicin Delivery and Preliminary Immune-Related Modulation in B16-F10 Melanoma
Source: Biomedicines. 2026 Jul 19;14(7):1624. doi: 10.3390/biomedicines14071624 (PMC13406409; doi:10.3390/biomedicines14071624)
Supplement: Supplementary file 1 [file biomedicines-14-01624-s001.zip › biomedicines-4420030-supplementary.pdf]

Supporting Information

A Self-Assembling Peptide Platform for Intratumoral Doxorubicin Delivery and Preliminary Immune-Related Modulation in B16-F10 Melanoma

Supplementary Methods

S1. Transmission electron microscopy (TEM)

ffky-antiCD3/DOX were prepared using the same formulation route as that used for the main study. The molar ratio was maintained at n(DOX):n(peptide) = 0.05. Samples were allowed to equilibrate at room temperature or 37 °C for 30–60 min before measurement. A droplet of sample was placed on a carbon-coated copper grid for 1–2 min, excess liquid was removed, and the grid was negatively stained with 1–2% phosphotungstic acid for 30–60 s. After air-drying, representative micrographs were collected under identical imaging conditions where possible.

S2. In vitro release of doxorubicin

The in vitro DOX release behavior was preliminarily evaluated using a dialysis method. Free DOX and ffky-antiCD3/DOX with the same DOX concentration were placed into dialysis bags and immersed in pH 6.5 buffer containing 10% FBS at 37°C under gentle shaking. At predetermined time points, release medium was collected and replaced with an equal volume of fresh prewarmed medium. DOX concentration was determined after protein precipitation, and cumulative release was calculated according to the standard curve prepared in the same serum-containing matrix.

Supplementary Figures and Captions

Figure S1. Representative TEM images of ffky-antiCD3/DOX assemblies.

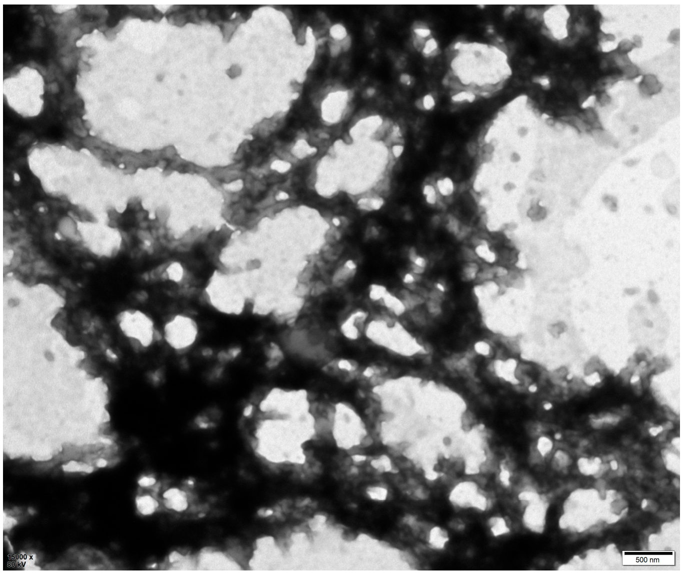

Figure S1. Representative TEM images of ffky-antiCD3/DOX assemblies. TEM images of negatively stained ffky-antiCD3/DOX showed heterogeneous irregular/network-like supramolecular assemblies rather than uniform spherical nanoparticles. The formulation was prepared at a molar ratio of n(DOX):n(ffky-antiCD3) = 0.05. Scale bars: 500 nm.

Figure S2. Preliminary in vitro DOX release behavior under tumor-mimicking acidic conditions.

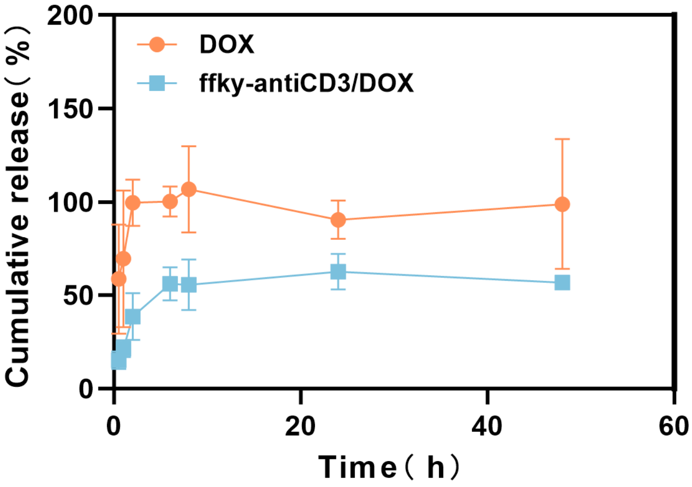

Figure S2. Preliminary in vitro DOX release behavior under tumor-mimicking acidic conditions. Cumulative release profiles of free DOX and ffky-antiCD3/DOX in pH 6.5 buffer containing 10% FBS at 37°C. The ffky-antiCD3/DOX formulation was prepared at a molar ratio of n(DOX):n(ffky-antiCD3) = 0.05. Data are presented as mean ± SD, $n = 3$.
